# Dual single-port laparoscopic surgery for metastatic low-grade serous ovarian carcinoma: a case report of an innovative minimally invasive approach adhering to no-touch isolation technique

**DOI:** 10.3389/fsurg.2026.1938536

**Published:** 2026-09-18

**Authors:** Xiaoqing Qing, Wen Zhou, Gang Liao, Dongqin Zhao

**Affiliations:** 1 Department of Anesthesiology, The First Affiliated Hospital of Chongqing Medical University, Chongqing, China; 2 Department of Nursing, The Second Affiliated Hospital of Chongqing Medical University, Chongqing, China; 3 Department of Gastrointestinal Surgery, The First Affiliated Hospital of Chongqing Medical University, Chongqing, China; 4 Department of Nursing, The First Affiliated Hospital of Chongqing Medical University, Chongqing, China

**Keywords:** case report, cytoreductive surgery, dual single-port laparoscopy, no-touch isolation technique, ovarian cancer, peritoneal metastases

## Abstract

The management of peritoneal metastases in young patients with ovarian cancer demands a delicate balance between oncological radicality and quality of life. We present the case of a 34-year-old woman with a final pathological diagnosis of primary advanced-stage (FIGO IIIC) low-grade serous ovarian carcinoma (LGSC) of the left ovary, with metastases to the sigmoid colon, pelvic peritoneum, vesical peritoneum, diaphragm, and omental surface. The diagnosis was confirmed by comprehensive immunohistochemistry (PAX8+, WT1+, ER+, PR+, CA125+, p53 wild-type) and complete histopathological examination. To strictly comply with the “no-touch” isolation principle while maximizing the advantages of minimally invasive surgery, we successfully performed a novel dual single-port laparoscopic cytoreductive procedure. The operation encompassed anterior resection, left salpingo-oophorectomy, omentectomy, and peritonectomy, all accomplished through two discrete 4–5 cm single-port incisions, Each platform housed three channels: one for the camera and two for working instruments, facilitating complex multivisceral and peritoneal resections. Complete macroscopic cytoreduction (R0) was achieved, with negative resection margins and only one positive lymph node among three harvested.This case demonstrates the technical feasibility and short-term safety of this innovative approach in a carefully selected patient with advanced ovarian cancer and peritoneal dissemination. However, the short follow-up period is a major limitation, and long-term oncological equivalence or superiority over standard approaches cannot be claimed. The purported advantages regarding reduced tumor manipulation, lower port-site metastasis risk, and superior recovery remain hypothetical and require validation in larger studies. We are continuing surveillance to assess longer-term outcomes.

## 1 Introduction

Ovarian cancer is one of the most lethal gynecologic malignancies, characterized by a propensity for peritoneal dissemination even in early-stage disease (1). Compared with high-grade serous carcinoma, low-grade serous carcinoma is less aggressive but often presents at an advanced stage with extensive peritoneal involvement, including metastases to the colon, pelvic and abdominal peritoneum, and omentum (2). For young patients, the dual therapeutic goals of achieving complete cytoreduction and preserving quality of life pose substantial clinical challenges.

Peritoneal metastases from ovarian cancer frequently involve multiple abdominal and pelvic compartments, necessitating multivisceral resections such as and extensive peritoneal stripping procedures (including omentectomy and peritonectomy). Traditional open surgery, while allowing for thorough exploration and tumor removal, is associated with significant trauma, prolonged recovery, and higher morbidity, while laparoscopic approaches could improve postoperative outcome (3). However, in cases of suspected disseminated intra-abdominal disease, concerns regarding port-site tumor seeding and inadequate tumor clearance often limit its application (4). Moreover, conventional multiport laparoscopy still requires several small incisions, each carrying a risk of tumor implantation and contributing to cumulative tissue trauma.

The “no-touch” isolation principle, originally described in colorectal oncology, is equally relevant in ovarian cancer surgery, where minimizing tumor manipulation and avoiding disruption of peritoneal deposits are critical to preventing cancer cell dissemination. Balancing this oncologic tenet with the advantages of minimally invasive surgery remains a key clinical dilemma (5). In this context, the dual single-port laparoscopic strategy represents an evolution in minimally invasive oncology surgery. By utilizing only two small access sites (typically 4–5 cm each), the total incision length is substantially shorter than in both open surgery and conventional multiport laparoscopy. This design not only reduces the number of potential ports for tumor seeding but also minimizes parietal trauma, potentially enhancing postoperative recovery while adhering to oncological safety. This innovative dual single-port strategy was specifically designed to achieve complete tumor cytoreduction while adhering strictly to the no-touch isolation technique. By utilizing only two discrete access points, strategically placed to address the pelvic and abdominal compartments separately, this design may potentially reduce the number of potential ports for tumor seeding and could be hypothesized to minimize parietal trauma, thereby potentially enhancing postoperative recovery. However, these theoretical advantages remain to be validated in larger prospective studies.

## 2 Case description

Our manuscript reporting adheres to Case report (CARE) guidelines (6). Written informed consent was obtained from the patient for publication of this case report and accompanying images.

As this study is a case—by—case analysis, it does not involve any interventional operations on patients, thus not increasing any additional risks for patients. Moreover, the patient information has been de—identified, which meets the circumstances of exemption from ethical review stipulated in the Measures for Ethical Review of Human—related Life Science and Medical Research [Guo Wei Ke Jiao Fa (2023) No. 4]. After confirmation by the hospital's ethics committee, this study can be exempted from ethical review. However, all forms of informed consent have been informed to the patients, and they have signed the relevant documents. Written informed consent was obtained from the patient for the publication of this case report and accompanying images. In addition to the standard surgical consent, the patient provided specific, written consent for the innovative dual single-port procedure, after a detailed explanation of its potential benefits, risks, and its non-standard nature compared to conventional open surgery.

### 2.1 General information

A 34-year-old unmarried woman with no significant past medical history presented after incidentally palpating a left lower abdominal mass approximately ten days prior. Upon admission, the patient was in good general condition, with no weight loss or obstructive symptoms. Physical examination revealed a palpable mass in the left lower quadrant, without signs of peritonitis. Vital signs were within normal limit. The patient expressed a strong desire to preserve future fertility potential. After multidisciplinary discussion, it was decided to preserve the uterus and the right adnexa, as they appeared free of disease on preoperative imaging and intraoperative inspection, while the left adnexa was resected due to obvious tumor involvement. This approach does not constitute standard fertility-sparing surgery (which is typically reserved for early-stage unilateral disease), but rather a compromise aiming to balance oncological radicality with reproductive wishes in this advanced-stage setting.

### 2.2 Diagnostic reasoning

Imaging studies (MRI and CT) revealed a solid mass at the rectosigmoid junction measuring approximately 6 cm in diameter, with surrounding enlarged lymph nodes and nodular lesions in the left paracolic gutter and pelvic peritoneum, highly suggestive of metastatic disease (Figure 1). Colonoscopy identified a cauliflower-like neoplasm at approximately 40 cm from the anal verge, partially obstructing the lumen (Figure 2). Biopsy of the lesion revealed a malignant tumor with papillary architecture and psammoma bodies, highly suggestive of metastatic carcinoma, possibly of ovarian or peritoneal serous origin. Subsequent immunohistochemistry supported a metastatic profile consistent with a primary ovarian low-grade serous carcinoma. Serum tumor markers (CA-125) were elevated, further supporting the ovarian origin. The final pathological workup (including a 17-marker immunohistochemical panel) confirmed the ovarian origin: CK7(+), PAX8(+), WT1(+), CA125(+), ER(+), PR(+), p53 wild-type (5% weak positivity), Ki-67 index 30-40%; CK20(−), CDX2(−), Villin(−), SATB2(+), CR(−), D2-40(−)，effectively ruling out a primary colorectal adenocarcinoma. These findings, together with the papillary architecture and psammoma bodies, established the diagnosis of low-grade serous carcinoma of ovarian origin (FIGO stage IIIC based on the involvement of the sigmoid serosa, pelvic peritoneum, vesical peritoneum, diaphragm, and left paracolic lymph node).

**Figure 1:**
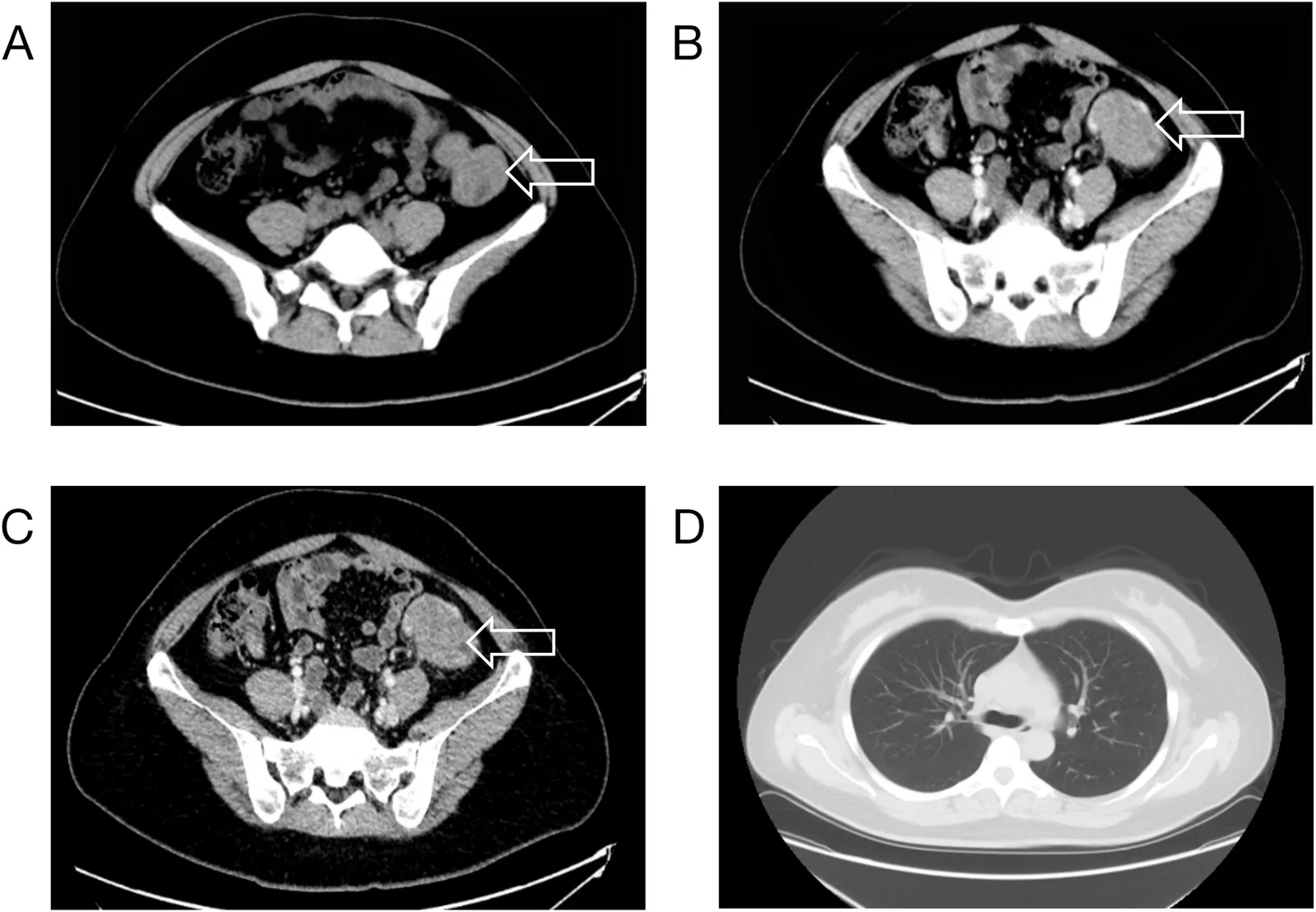
**(A)** the enhanced arterial phase of the abdomen (at the tumor site), **(B)** the enhanced venous phase of the abdomen (at the tumor site), **(C)** the pelvis (at the tumor and nodule sites), **(D)** the plain abdominal scan (at the tumor site).the white arrow points to the location of the tumor.

**Figure 2:**
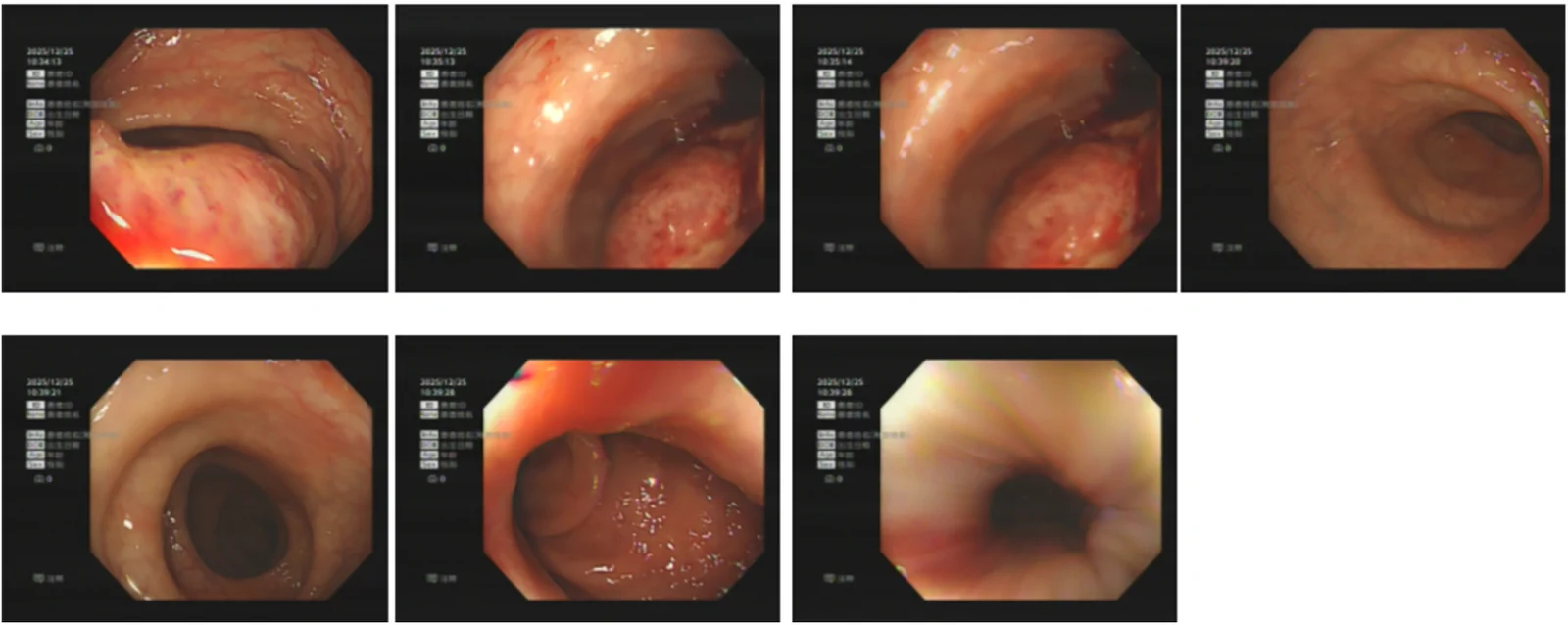
The colonoscopy revealed a cauliflower-like neoplasm extending around the circumference at a position approximately 40 cm from the proximal end of the sigmoid colon and adjacent to the descending colon. The lesion was located at the bend and the colonoscope could not pass through.

### 2.3 Diagnostic challenges

Given the extent of disease—involving the sigmoid colon, left adnexa, omentum, and peritoneal surfaces (pelvic, vesical, and diaphragmatic)—the primary challenge lay in selecting the optimal surgical approach. While open laparotomy would allow en-bloc resection and reduce the risk of tumor spillage, it would entail a large abdominal-pelvic incision with considerable morbidity and slow recovery. Conventional multiport laparoscopy, though minimally invasive, raised concerns about potential tumor cell implantation at multiple trocar sites, a risk emphasized by the gynecologic oncology team. The challenge was to reconcile the imperative of minimizing tumor manipulation and dissemination (the “no-touch” principle) with the desire to offer a minimally invasive, enhanced recovery pathway.

### 2.4 Multidisciplinary decision-making

The case was discussed in a multidisciplinary tumor board involving gastrointestinal surgeons, gynecologic oncologists, radiologists, and pathologists. After thorough review, the team proposed a novel dual single-port laparoscopic strategy. The rationale was to utilize only two small access sites, strategically placed to address the pelvic and abdominal compartments separately. The plan involved placing one single-port platform through a 4–5 cm infra-umbilical incision to address pelvic structures (left adnexa, pelvic peritoneum), and another through a supra-umbilical incision to manage the abdominal components (sigmoid colon, omentum, diaphragmatic peritoneum). This design aimed to minimize instrument passage and tissue manipulation across compartments, thereby respecting the no-touch isolation technique, while preserving the benefits of minimally invasive surgery. The criteria for considering this patient suitable for the dual single-port approach included: (1) young age and good performance status (ECOG 0), allowing tolerance of prolonged pneumoperitoneum; (2) disease burden amenable to two access sites — preoperative imaging suggested multi-compartment involvement (pelvis, sigmoid, omentum, diaphragm) but no diffuse miliary carcinomatosis; (3) absence of extensive upper-abdominal disease that would typically require laparotomy (e.g., massive liver surface or porta hepatis involvement). The patient was fully informed of the innovative nature of the procedure and provided written informed consent.

### 2.5 Treatment and outcome

After the induction of general anesthesia, the patient was placed in the lithotomy position. A 12 mmHg CO_2_ pneumoperitoneum was established via an umbilical incision, and a diagnostic laparoscopy was performed to confirm the absence of unexpected peritoneal dissemination. Based on the preoperative plan, two single-port devices were then placed: one at the infra-umbilical site and a second in the supra-umbilical midline (Figure 3). Each single-port platform contained three channels: one for the 5 mm 30° laparoscope and two for the working instruments (e.g., ultrasonic scalpel, bipolar forceps, and bowel graspers). This configuration allowed for independent instrument manipulation within each compartment.

**Figure 3:**
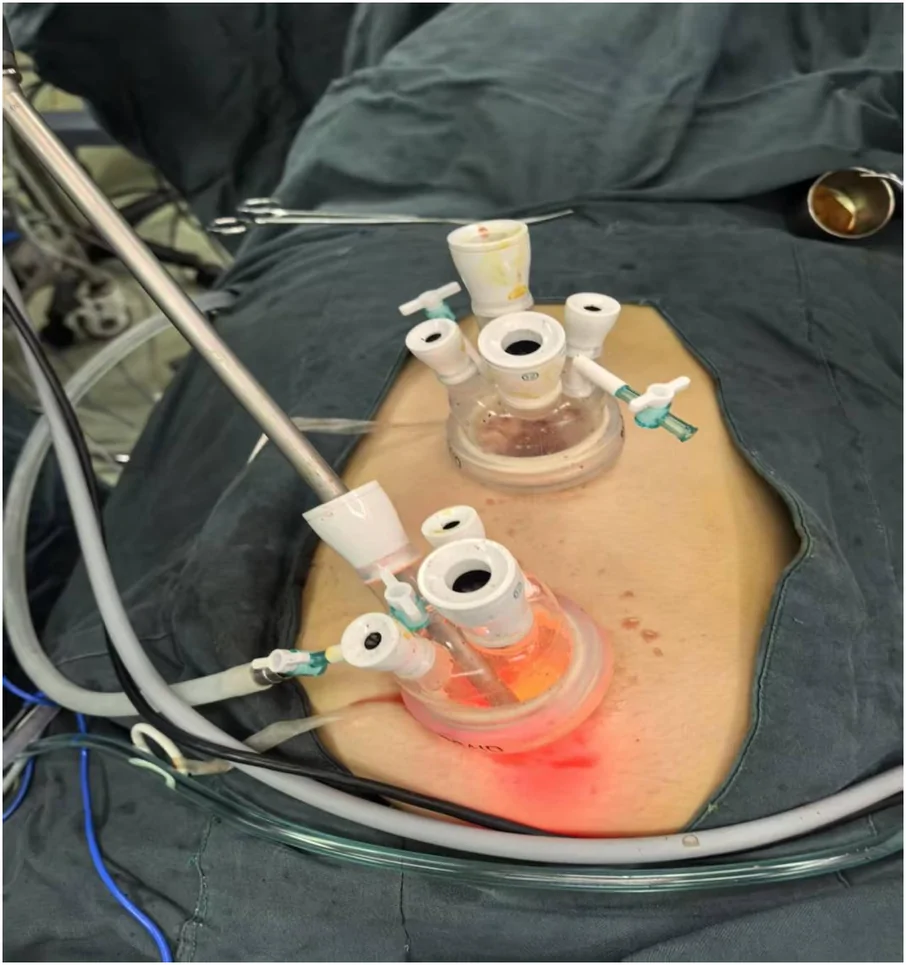
Double and single-port laparoscopic approach.

The surgical procedure proceeded in a systematic, compartment-based fashion. First, with the patient in a steep Trendelenburg and right-tilted position, the infra-umbilical port was utilized for the pelvic phase. After the gynecologic oncologist confirmed a suspicious nodule on the left ovarian surface, a left salpingo-oophorectomy was performed, with the specimen sent for frozen section (returned positive for atypical cells). A pelvic peritonectomy was then meticulously carried out, excising all visible tumor deposits from the pelvic, vesical, and rectouterine peritoneum. Careful preservation of the inferior hypogastric plexus and pelvic nerves was achieved through sharp dissection in the avascular retroperitoneal planes.

Attention was then turned to the abdominal phase via the supra-umbilical single port. The sigmoid colon was retracted medially, and the white line of Toldt was incised to develop the avascular plane between the mesocolon and Toldt's fascia. The left ureter and gonadal vessels were identified and preserved. The inferior mesenteric artery (IMA) was traced to its root, and a D3 lymphadenectomy was performed. The D3 lymphadenectomy was performed as an integral component of the formal oncological sigmoid colectomy, which was necessary to achieve an R0 resection for the colonic component of the disease. This does not represent a standard procedure for ovarian cancer but was dictated by the colorectal surgical principles applied to the involved bowel segment. The left colic artery was preserved, and the IMA was divided with clips. The inferior mesenteric vein was divided at a similar level. Dissection proceeded cranially along Toldt's fascia to mobilize the descending colon and splenic flexure, ensuring a tension-free anastomosis. Distally, the mesorectum was sharply dissected along the holy plane to expose the extraperitoneal rectum, with continuous visualization of the pelvic nerves. The rectum was divided using a linear stapler at a point at least 5 cm distal to the tumor. Following the vascular control and mobilization, a complete omentectomy was performed by separating the greater omentum from the transverse colon and dissecting it off the gastroepiploic arcade. Visible peritoneal deposits on the diaphragm were also excised.

Following complete cytoreduction, the pneumoperitoneum was temporarily released. The supra-umbilical incision was extended to allow for exteriorization of the bowel with a wound protector. The mobilized colon and tumor were delivered, and the proximal bowel was divided at a point at least 10 cm proximal to the tumor. The anvil of a circular stapler was secured in the proximal colon with a purse-string suture, and the bowel was returned to the abdominal cavity. The incision was partially closed around the single port, and the pneumoperitoneum was re-established. A tension-free, end-to-end anastomosis was created under laparoscopic guidance using the circular stapler introduced transanally. The anastomosis was inspected, found to be intact and well-vascularized, and reinforced with a barbed suture.

The abdominal and pelvic cavities were thoroughly irrigated with copious normal saline. Given the extensive peritoneal disease and complete cytoreduction, and supported by emerging evidence suggesting a potential benefit of HIPEC in low-grade serous carcinoma, the decision was made to proceed with Hyperthermic Intraperitoneal Chemotherapy (HIPEC). Four drainage catheters were placed through the abdominal wall in the bilateral upper and lower quadrants and connected to a closed-circuit perfusion system (Figure 4). After confirming meticulous hemostasis, the incisions at both single-port sites were closed in layers. The total operative time was 6 h and 14 min, with an intraoperative blood loss of only 50 mL. The patient remained hemodynamically stable throughout.

**Figure 4:**
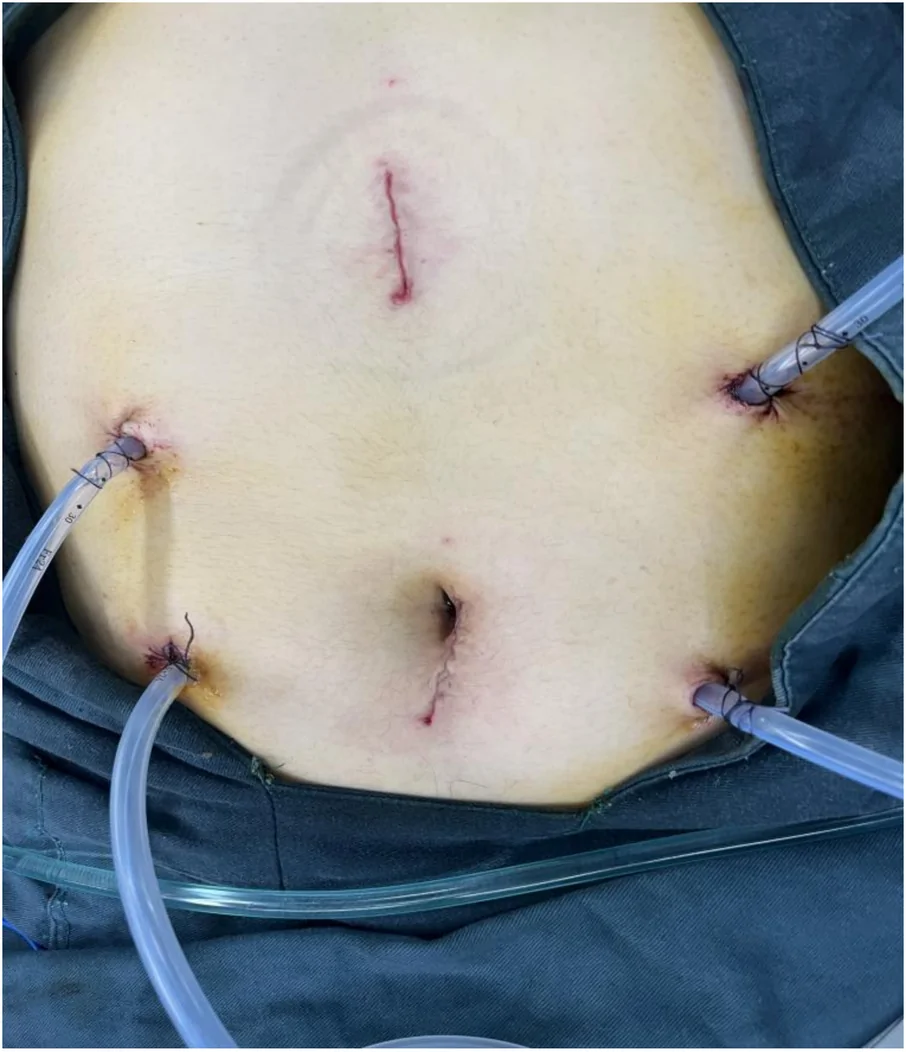
Postoperative incision and HIPEC perfusion tube.

The patient was extubated in the operating room and transferred to the ward in stable condition. The postoperative course was uneventful. The surgical drains were utilized for the subsequent HIPEC procedure as planned. The HIPEC regimen consisted of paclitaxel 275 mg and cisplatin 135 mg in normal saline, administered in three consecutive cycles through a closed-circuit perfusion system (total perfusion time 60 min, inflow temperature 42  °C). This regimen was selected based on institutional protocol and the recommendations of the gynecologic oncology team. The patient was discharged on postoperative day 10 in good condition. While the postoperative recovery was uneventful, the hospitalization duration was influenced by the planned HIPEC monitoring, the standard recovery pathway for a multivisceral resection, and the patient's preference for a full recovery period before discharge. Pathological analysis confirmed primary low-grade serous carcinoma of the left ovary with metastases to the sigmoid colon (invading from serosa to mucosa), omentum, pelvic peritoneum, vesical peritoneum, and diaphragm. All surgical margins (proximal and distal colonic margins, peritoneal resection margins) were negative for tumor. Lymph node dissection yielded three regional lymph nodes, of which one showed metastatic carcinoma (1/3). No macroscopic residual disease was observed after cytoreduction (R0 resection). The left fallopian tube showed focal intraluminal tumor emboli, but the right adnexa and uterus were grossly uninvolved and preserved. Final FIGO stage (2018): IIIC (T3c, N1, M0) due to peritoneal involvement beyond the pelvis and retroperitoneal lymph node metastasis. At 3-month follow-ups, the patient had completed rehabilitation, presented with a good performance status, and whole-body CT scans showed no evidence of local recurrence. She was then referred to the gynecologic oncology clinic for initiation of adjuvant systemic therapy based on the post-operative pathology results and genetic testing.

## 3 Discussion

The management of peritoneal metastases from ovarian cancer remains a formidable clinical challenge, necessitating a balance between oncological radicality and postoperative recovery (7). In this case, the young patient presented with extensive intra-abdominal disease involving the sigmoid colon, left adnexa, omentum, and multiple peritoneal surfaces. Without surgical intervention, progressive obstruction, ascites, and cancer-related symptoms would inevitably impair her quality of life and survival. Thus, an aggressive cytoreductive approach was indicated, aiming to achieve complete tumor removal and alleviate symptom burden.

Given the extent of disease, the central dilemma lay in selecting the appropriate surgical approach. Open laparotomy, while allowing thorough exploration and en-bloc resection, would require a large abdominopelvic incision associated with significant pain, prolonged ileus, and delayed recovery—particularly undesirable in a young, otherwise healthy patient. Conversely, conventional multiport laparoscopy, though minimally invasive, raised valid concerns regarding potential tumor seeding at trocar sites, a risk emphasized by the gynecologic oncology team (8). In this context, the dual single-port laparoscopic technique was conceived as a novel solution to reconcile the imperatives of “no-touch” oncology and enhanced recovery.

The rationale for adopting this innovative approach rested on two pillars: adherence to no-touch isolation technique and minimization of surgical trauma. By utilizing only two discrete access points, strategically placed to address the pelvic and abdominal compartments separately, the procedure inherently limited instrument passage and the need to maneuver specimens across the entire cavity. This compartmentalized approach directly reduced opportunities for tumor cell dissemination.

Moreover, the small incisions translated into less postoperative pain, earlier mobilization, and a potentially shorter hospital stay, aligning with modern enhanced recovery after surgery (ERAS) protocols (9, 10). The successful execution of the procedure, with complete resection of all visible lesions and minimal blood loss (50 mL), demonstrates that meticulous preoperative planning and advanced laparoscopic skills can extend the boundaries of minimally invasive cancer surgery. We acknowledge that the term “fertility-sparing” may be misleading in this context; the procedure preserved only one ovary and the uterus, which may offer a possibility of future pregnancy via assisted reproductive techniques, but the oncological safety of this strategy in advanced LGSC remains unproven and requires careful patient selection and counseling.

Although HIPEC is most extensively studied in high-grade serous and primary peritoneal cancers, emerging evidence suggests potential benefit in low-grade serous carcinoma as well. A recent propensity-matched analysis of the National Cancer Database (NCDB) demonstrated that among patients with LGSC undergoing cytoreductive surgery, the addition of HIPEC was associated with significantly improved overall survival (log-rank *χ*²=3.934, *p* = 0.047). Additionally, a single-center series reported favorable outcomes in rare histological subtypes (including LGSC) treated with CRS + HIPEC, particularly in younger patients (age <65 years) and those with PCI ≤20. These data, although retrospective, provided the rationale for our intraoperative decision to proceed with HIPEC after complete cytoreduction. Nevertheless, we acknowledge that the evidence remains limited and the decision must be individualized.

It is important to note that HIPEC may adversely affect ovarian reserve due to the thermal and chemotherapeutic injury to pelvic tissues. The patient was fully informed of this potential risk prior to the procedure. The decision to proceed with HIPEC while preserving the right ovary was made after careful multidisciplinary deliberation, weighing the potential fertility benefit against the oncological benefit of complete cytoreduction followed by HIPEC. This balance was particularly relevant given the patient's young age and strong reproductive wishes, and the final decision was made with her explicit informed consent. However, the long-term fertility outcomes and oncological safety of this individualized approach remain uncertain and require further follow-up.

The patient was discharged on postoperative day 10. This is presented as a descriptive observation of the actual clinical course, not as evidence of superiority over open or other minimally invasive approaches, particularly given the confounding factors of the HIPEC procedure and the patient's personal preference for a full recovery period before discharge

## 4 Limitations

This is a single-case report, and the purported advantages of the dual single-port technique in reducing tumor manipulation, dissemination, port-site metastasis, and surgical trauma remain hypothetical and cannot be substantiated by this isolated experience. The short follow-up period (3 months) is insufficient to draw any conclusions regarding oncological safety, recurrence patterns, or superiority over conventional approaches. Moreover, the selection of laparoscopic surgery in the setting of extensive peritoneal disease is controversial, and our favorable outcome does not warrant generalization. Long-term prospective studies with larger cohorts are urgently needed to validate this technique and to define patient selection criteria.

## 5 Conclusions

This case demonstrates that in a highly selected patient with peritoneal metastases from ovarian cancer, a carefully designed dual single-port laparoscopic strategy is technically feasible and appears safe in the short term. We underscore the importance of tailoring the surgical approach to the individual patient's disease extent and functional status. However, we explicitly acknowledge that no claims of accelerated recovery, reduced morbidity, or superiority over standard approaches can be made based on this single case. The postoperative course (discharge on postoperative day 10) is presented as a descriptive observation rather than as evidence of comparative benefit. Future comparative studies and technical refinements will be necessary to define the role of this innovative technique in the management of advanced ovarian cancer.

## Data Availability

The original contributions presented in the study are included in the article/Supplementary Material, further inquiries can be directed to the corresponding author.
